# The Role of Short-Chain Fatty Acids in the Interplay between a Very Low-Calorie Ketogenic Diet and the Infant Gut Microbiota and Its Therapeutic Implications for Reducing Asthma

**DOI:** 10.3390/ijms21249580

**Published:** 2020-12-16

**Authors:** Naser A. Alsharairi

**Affiliations:** Heart, Mind & Body Research Group, Menzies Health Institute Queensland, Griffith University, Gold Coast 4222, Australia; naser.alsharairi@gmail.com

**Keywords:** ketogenic diet, asthma, SCFAs, infant gut microbiota, pregnancy, lactation

## Abstract

Gut microbiota is well known as playing a critical role in inflammation and asthma development. The very low-calorie ketogenic diet (VLCKD) is suggested to affect gut microbiota; however, the effects of VLCKD during pregnancy and lactation on the infant gut microbiota are unclear. The VLCKD appears to be more effective than caloric/energy restriction diets for the treatment of several diseases, such as obesity and diabetes. However, whether adherence to VLCKD affects the infant gut microbiota and the protective effects thereof on asthma remains uncertain. The exact mechanisms underlying this process, and in particular the potential role of short chain fatty acids (SCFAs), are still to be unravelled. Thus, the aim of this review is to identify the potential role of SCFAs that underlie the effects of VLCKD during pregnancy and lactation on the infant gut microbiota, and explore whether it incurs significant implications for reducing asthma.

## 1. Introduction

Low carbohydrate diets (LCDs) can be highly heterogeneous in terms of carbohydrate (CHO) content and quality, with no consensus on its precise definition [1], and for this reason it is difficult to interpret comparisons of results between studies. The very low-calorie ketogenic diet (VLCKD), a popular type of LCD, is similar to the modified Atkins regime in terms of restricting CHO while emphasizing a high-fat regimen [2]. As the VLCKD seems to be an area of growing interest in preventing and treatment of several diseases [3,4,5,6,7,8], evidence of its effect on the gut microbiota is inadequate and still ongoing in animal models and humans [9]. In fact, thevery low-calorie diet (VLCD) contributes to gut microbiota remodelling in humans [10], and “keto microbiota,” which refers to a gut microbiota shaped by a ketogenic diet (KD), and may play a major role in enhancing the response of the host to therapy [11]. The low CHO, adequate protein and high-fat KD has been found to be associated with increased beneficial gut microbiota-related profiles including *Bacteroidetes* phylum in children with refractory epilepsy. However, this increase occurs with respect to reducing the overall microbial diversity, probably due to the low CHO content of the diet, which can disrupt the abundance of other beneficial microbiota responsible for degrading complex CHO [11].

The symbiotic relationship that has evolved between humans and their gut microbiota provides several benefits for humans, including regulating host immunity, producing vitamins K and B, protecting against pathogens, strengthening gut integrity and producing metabolites such as short chain fatty acids (SCFAs) [12]. The composition of the infant gut microbiota is driven by several factors, such as mode of delivery and feeding, maternal antibiotic use and nutrition and body mass index (BMI) [13]. The stability of the gut microbiota, reached between 2 to 18 years of age, is varied by phylum, with *Bacteroidetes* exhibiting the highest temporal stability [12].

The maternal gut microbiota is an extremely dynamic entity influenced by several perinatal factors, including diet, which may in turn influence the infant gut microbiota composition [13]. For this reason, establishing the influence of specific restricted dietary patterns such as VLCKD on the infant gut microbiota composition is of substantial additional importance. This pattern may positively or negatively influence the gut microbiota composition and its related effects on host health [9]. Different types of the KD exist (including standard, cyclical, targeted and high protein KD), but standard KD (VLCKD) is considered a highly restricted CHO diet [14]. The VLCKD is an extremely low CHO, high fat and moderate protein diet [15], which restricts CHO to less than 50 g per day [14,15,16], with a ratio of macronutrients being 70% from fat, 20% from protein and 10% from CHO [14]. The source of CHO (dietary fiber), fats (high in polyunsaturated fatty acid (PUFA), moderate in monounsaturated fatty acid (MUFA) and low in saturated fatty acid (SAT)) and plant-based protein should be highly considered when planning a VLCKD regimen, which plays a key role in shaping the function/composition of gut microbiota and producing SCFAs [17,18,19]. The VLCKD can lead to a metabolic state called “ketosis”, which results in increased liver ketone bodies (KBs) production [20], and may in some cases (type 1 diabetes, gestational diabetes) lead to a pathological state called diabetic ketoacidosis (DKA), where too many KBs accumulate in the blood, causing it to become highly acidic [21]. 

Oxidative stress is an important feature of airway inflammation in asthmatic children [22]. It is hypothesized that β-hydroxybutyrate (βOHB), a major component of KBs, is significant in reducing oxidative stress by inhibiting reactive oxygen species (ROS)/superoxide production and improving mitochondrial activity [23,24]. It has also shown anti-inflammatory effects by inhibiting the leucine-rich-containing family, pyrin domain-containing-3 (NLRP3) inflammasome-mediated inflammatory chronic disease [20,23]. Therefore, it is particularly important to determine whether adherence to a VLCKD during pregnancy and lactation has a beneficial effect on childhood asthma. Indeed, the clinical relevance of such effects is yet to be investigated. Changes in the gut microbiota are associated with various pathological states, and it has been suggested that imbalance of the gut microbiota (dysbiosis) increases the risk of developing asthma later in life [25]. Gut dysbiosis is characterized by increased levels of *Proteobacteria* and decreased levels of *Veillonella*, *Lachnospira*, *Rothia*, *Roseburia* and *Faecalibacteria* in asthmatic children [26,27]. The VLCKD can significantly change the gut microbiota composition of pediatric patients [9], suggesting that gut microbiota should be taken into consideration as a potential alternative therapeutic treatment for asthma. The VLCKD therapeutic effect may result from metabolic reprogramming and epigenetic markers as mechanisms with gut metabolites [28,29], where all may be involved in altering the infant gut mictobiota, thereby reducing the risk of asthma. The exact mechanisms underlying the effect of VLCKD in pregnancy and lactation against childhood asthma are still largely unknown. Therefore, this review aims to provide an overview of whether the VLCKD use influences the infant gut microbiota composition and the protective effects thereof on asthma. Herein, the paper highlights the role of gut metabolites SCFAs as potential mechanisms that may underlie these effects.

## 2. Methods

A non-systematic search of the published literature is conducted between 1 January 2000, and 30 November 2020, in the PubMed database using the following keywords: KD, KBs, asthma, pregnancy, lactation/breastfeeding, SCFAs, epigenetic, gut inflammation, pro-inflammatory cytokines and the infant gut microbiota. Searches include reviews/systematic reviews, meta-analysis, randomized controlled trials (RCTs)/experimental studies and observational studies (case-reports, cross-sectional, case-control, cohort) published in English.

## 3. Ketone Body Metabolism

The main metabolic pathways for ketone body metabolism include ketogenesis and ketolysis. Adherence to KD causes the body to enter the ketogenesis pathway to produce three main KBs: βOHB, acetoacetate (ACA) and acetone (least abundant) [20]. Ketogenesis takes place in the mitochondrial matrix of hepatocytes, where free fatty acids (FFAs) are released from adipose tissue during lipolysis under low insulin conditions, along with stimulating catecholamines, cortisol, glucagon and growth hormone secretion. FFAs are broken down via β-oxidation to acetyl-coenzyme A (acetyl-CoA), which is used as a precursor for the production of βOHB and ACA [20,30]. These are released into the circulation for use in extrahepatic tissues via the monocarboxylate transporter 1 (MCTI1), where the ketolysis process takes place. Once taken up by target tissues, βOHB is transformed to ACA via βOHB dehydrogenase (βDH) and ACA is transformed back to acetyl-CoA viaβ-ketoacyl-CoA transferase (βCT). Acetyl-CoA then goes through a thetricarboxylic acid (TCA) cycle to generate nicotinamide adenine dinucleotide (NADH) and flavin adenine dinucleotide (FADH2) via the oxidative phosphorylation pathway to produce adenosine triphosphate (ATP) [20,31]. The ketogenesis and ketolysis pathways are also active during starvation/fasting [20,24,32,33], and the periods of pregnancy and childbirth [24,34], where CHO availability is significantly diminished, or fatty acid levels are increased.

## 4. Ketone Bodies as Epigenetic Modifiers in Asthma

Epigenetic changes constitute the key regulator of gene expression and cellular metabolism, and their dysregulation may contribute to several diseases [35], including childhood asthma [36], where changes may start in utero following prenatal environmental exposures (e.g., maternal smoking, allergen, dietary supplements) or during early life [37]. Epigenetic changes in breastfed infants, particularly changes in DNA methylation patterns, may be influenced by breastfeeding, but further studies are needed to explore the role of epigenetic mechanisms in the associations between breastfeeding and asthma [38]. DNA methylation, non-coding RNA and histone modifications are the most common epigenetic mechanisms existing in childhood asthma, which can regulate gene expression through effects on chromatin structure and contribution to gene silencing [39,40].

Epigenetic changes are influenced by KBs [11], and the βOHB not only regulates cellular processes such as signaling metabolites [41], but also influences the gut microbiota and increases butyrogenesis [42], in which epigenetic mechanisms are involved [43,44]. Ketosis has been linked to epigenomic reprogramming and displays as covalent KB-induced histone post-translational modifications, including histone methylation (Kme), histone/lysine acetylation (Kac) and β-hydroxybutyrylation (Kbhb), which regulate chromatin architecture and gene expression during adherence to KD, DKA and fasting ketosis [45]. Kac and Kbhb consider the key epigenetic mechanisms for activation of βOHB to modulate immune cell function and inflammation [46]. The βOHB, an endogenous histone deacetylase (HDACs) inhibitor, has a well-known protective role against oxidative stress. In animal models, adherence to KD, which increases βOHB levels, is associated with increased histone Kac at the promoter regions of the forkhead box (Foxo3a) and metallothionein 2A (Mt2), which targets oxidative stress resistance genes activated by HDAC class I and II inhibitors [45,46,47]. In response to high levels of βOHB, histone Kbhb levels with site-specific lysine residues (H3K4, H4K8, H3K9, H4K12, H3K56) are elevated significantly in human embryonic kidney 293 (HEK293) cells during prolonged fasting, suggesting that lysine Kbhb at these residues regulates chromatin structure and functions [43]. HEK293 cells are found to transiently transfect with ORM (yeast)-Like protein isoform 3 (ORMDL3) mRNA expression, an asthma susceptibility gene located on chromosome 17q21 in children [48]. ORMDL3 suppresses the sarco-endoplasmic reticulum Ca^2+^ pump (SERCA) leading to a decreased endoplasmic reticulum (ER) Ca^2+^ concentration and activating unfolded-protein response (UPR) signaling pathway [49]. This pathway can induce increased expression of chemokines, metalloproteases and activating transcription factor (ATF6) in lung epithelial cells, which are involved in the pathogenesis of asthma [50]. βOHB suppresses inflammation via inhibition of protein expression of ER stress response pathway (known as UPR). It also enhances both Foxp3 and manganese superoxide dismutase (MnSOD) transcription through AMP-activated protein kinase (AMPK) activation, a cellular energy sensor which regulates energy homeostasis, leading to a reduction in the level of cellular oxidative stress [51]. This suggests that βOHB may regulate histone Kbhb and protect HEK293 cells against oxidative stress via suppressing ER stress. Taken together, βOHB acts as a potent epigenetic modifier and exerts its anti-inflammatory effect providing potential targeted therapy in asthma through mechanisms for epigenetic regulation.

## 5. Short-Chain Fatty Acids: A Link between Maternal VLCKD, the Infant Gut Microbiota and the Potential Role in Reducing Asthma

The link between VLCKD during pregnancy and lactation, the infant gut microbiota and the potential role in reducing asthma is still unknown. Exposure to antenatal and postnatal factors (e.g., maternal nutrition, antibiotic exposure, maternal microbiota, skin contacts, maternal smoking and mode of delivery and feeding), which are known triggers of asthma, also have the potential to alter DNA and histone methylation of several genes [28,52,53], resulting in altered lung function in the offspring [52]. Gut microbial metabolites have been considered as substrates/cofactors for mediating the relationship between host epigenetic markers and alterations in gut microbiota composition [54]. The gut microbiota produced SCFAs as epigenetic cofactors/substrates, which induce changes of DNA methylation and histone modifications [28,29], and thereby, have potential value as underlying mechanisms. Maternal nutrition during pregnancy and lactation plays a significant role in modulating the infant gut and milk microbiota composition [13]. However, how maternal diet, particularly VLCKD, epigenetically determines asthma phenotypes in the next generation of offspring is still undetermined. Importantly, it would be interesting to understand how the VLCKD during pregnancy and lactation, as a result of the influence of SCFAs, can modulate the infant gut microbiota profile, which could in turn lead to specific epigenetic changes, resulting in a modified risk of asthma in offspring.

Several studies showed that maternal diet during pregnancy, particularly the macronutrient composition of VLCKD (SFA, omega-3 PUFA, linoleic (ω-6) fatty acid, vegetable protein) and the dietary patterns characterized by high intake of these nutrients, may promote SCFAs production through diverse microbiota in the maternal and infant gut [13,55,56,57,58,59,60,61]. Breast milk feeding of many different microbial species may explain the increased abundance of these species in the infant gut and vice versa, suggesting vertical transmission during breastfeeding via the entero-mammary pathway. This pathway involves the passage of diverse microbes from the lumen of the mother’s gut by dendritic cells (DCs)/macrophages during late pregnancy, and subsequently, translocates them to the lactating mammary glands through the lymphatic system and/or blood stream [62,63]. The gut microbiota is able to produce SCFAs in breast milk, which can pass through the maternal gut into the mammary gland via the systemic circulation [64], suggesting SCFAs may transfer to the infant gut through the breast milk. Dietary macronutrient intake in the VLCKD during lactation may produce SCFAs and influence the infant gut SCFA-producing bacteria by influencing the breast milk microbiota [13].

### 5.1. Short-Chain Fatty Acids

The SCFAs, acetate, propionate and butyrate (in a molar ratio at 3:1:1), constitute the dominating microbial fermentative end-products of undigested/unabsorbed dietary CHO (mainly fiber and resistant starch), and to a relatively minor extent, endogenous proteins (peptides and amino acids) in the colon [65,66]. Acetate is the most dominant circulating metabolite in the feces of breastfed infants, followed by propionate and butyrate [67,68]. Lactate is a major intermediate metabolite present in high levels in the feces of infants [67,68], where it acts with acetate as substrates for butyrate production by certain enteric bacterial species [67]. Fecal acetate is accumulated in high levels in the colon of adults, whereas lactate is detected at low levels, as they may act as a substrate for butyrate and propionate-producing bacteria [66,67]. Diets high in resistant starch are linked to an increase in the fecal butyrate levels in healthy adults [69]. SCFAs driven by maternal gut microbiota are also likely detected in breast milk. A few studies have revealed that butyrate and acetate are the most abundant SCFAs in breast milk [64,70,71]. Butyrate and acetate levels in breast milk are found to be significantly lower in atopic than non-atopic women [64].

SCFAs are mainly produced by the *Firmicutes* phylum. Butyrate is produced by acetate and/or lactate-utilizing butyrate-producing bacteria through the butyryl-CoA: acetate CoA-transferase pathway [65,72,73]. This pathway is typically present in Lachnospiraceae (including *Eubacterium hallii, Eubacterium rectale*, *Roseburia intestinalis*, *Coprococcuscatus*) and Ruminococaceae (including *Faecalibacterium prausnitzii*) within the phylum *Firmicutes*, in which acetyl-CoA and butyrate are formed from butyryl-CoA and the transformation of the CoA moiety to the external acetate molecule [66,72,73]. The *Clostridium* spp. belonging to the *Firmicutes* phyla are also able to produce butyrate through the butyrate kinase pathway [66,74]. Other probiotic bacteria, such as *Bifidobacterium* and *lactobacillus* spp., utilize lactate and produce SCFAs, which exert anti-inflammatory effects in immune cells through an epigenetic mechanism such as butyrate-associated HDAC inhibition [53].

SCFAs have emerged as significant mechanisms linking diet, gut microbiota and the pathogenesis of asthma [65], and thereby, have become a significant therapeutic target for inhibiting lung pro-inflammatory responses [75]. SCFAs play a significant role in regulating host immune homeostasis, which are essential for accumulation and/or differentiation of the colonic regulatory T cells (T_regs_) [76,77]. Defects in T_regs_ function contribute to the failure of its ability to suppress an excessive Th2 response, which may promote the development of allergic asthma [78]. Butyrate acts as an HDAC inhibitor via the activation of G-protein coupled receptors (GPCRs) and FFA receptors (FFA2, FFA3) to enhance T_regs_ differentiation and DCs [79,80,81,82]. Butyrate is able to ameliorate gut inflammation by blocking nuclear factor-κB (NF-κB) activation in intestinal B-lymphocytes and induce regualtion of peroxisome proliferator-activated receptor gamma(PPARγ) expression [53].The NF-κB transcription factor binds to the inhibitor of kappa B (IκB) proteins, which is phosphorylated by kappa B kinase (IKK), in particular IKK-β/IKK-2, via the canonical pathway, leading to the enhancement of its transactivation potential in lung epithelial cells, where it affects inflammatory genes, leading to asthma [83,84]. Butyrate is considered an anti-allergic asthma treatment, which suppresses pro-inflammatory cytokines secretion and GATA binding protein 3 (GATA3) expression in the human pulmonary Group 2 Innate lymphoid cells (ILC2s) [82]. ILC2s play a role in the production of pro-inflammatory cytokines, and overexpression of the transcription factor GATA3 increased the expression of stimulation-expressed gene 2 (ST2) and thymic stromal lymphopoietin (TSLP) receptors in the human pulmonary ILC2, leading to the development of asthma [85,86].

SCFAs produce a strong synergistic effect with VLCKD in inducing ketosis [87]. The combination of βOHB-butyrate in relation to ketogenic initiatives is sensible [87]. The VLCKD is characterized by reducing the proportion of dietary CHO to a certain level [3,14,15], which may result in lowering the amount of dietary fiber intake [88]. This decrease in dietary fiber has the greatest potential to cause dysbiosis of the infant gut microbiota, which is associated with reduced butyrate-producing species, including *R. intestinalis* and *F. prausnitzii*. This ultimately leads to a weakened immune response and potentially contributes to asthma [89]. There is some evidence suggesting maternal dietary fiber intake or high acetate levels during pregnancy may reduce the risk of asthma in offspring. In one experimental study, a high-fiber diet during late pregnancy is shown to promote gut species belonging to the *Bacteroidetes* phylum, which produces high levels of acetate. Acetate suppresses estalished allergic airways disease in offspring by inhibiting HDACs (mainly HDAC9), leading to Foxp3 transcription, which enhances T_regs_ number and function. Foxp3 binds upstream of the fetal lungnitrate reductase catalytic subunit (NAPA) gene located on chromosome 1p36 to inhibit atrial natriuretic peptide (ANP) production [90], a molecule associated with asthma [91]. Another experimental study found that, compared with butyrate and propionate, high fecal SCFA acetic acid levels are associated with dietary fiber intake during pregnancy and reduced asthma risk with allergic sensitization [92].

### 5.2. SCFA-Producing Bacteria: A Potential Regulatory Role in Reducing Asthma

A review of the evidence shows that tumor necrosis factor (TNF-α), interferons (IFN-α/INF-β) and interleukins (IL-1α/IL-1β, IL-4–IL-6, IL-10, IL-13, IL-15–IL-20, IL-25, IL-31–IL-33) play a key role in the pathogenesis of allergic asthma [93]. This section presents SCFA-producing bacteria, and their potential therapeutic implications for reducing asthma.

#### 5.2.1. *Bifidobacterium* spp.

Bifidobacteria, referred to as Human-Residential Bifidobacteria (HRB), are Gram-positive anaerobic non-motile bacteria with highguanine-plus-cytosine (GC) content, which belong to the species *Bifidobacterium* within the Actinobacteria phylum [94,95]. Diets enriched in plant protein, unsaturated fat/low fat, high fiber/resistant starch and polyphenols increase gut abundance of Bifidobacteria [96]. Infants with a low relative abundance of *Bifidobacterium* are at higher risk of developing asthma later in life [97]. Evidence from human RCTs/experimental studies showed that the presence of *Bifidobacterium* spp. and strains resulted in regulated immune responses and improved the production of anti-inflammatory cytokines in infants, suggesting that *Bifidobacterium* could be of great potential as a therapeutic approach for reducing asthma. *B. longum* subsp. *infantis* has been shown to display anti-inflammatory properties through the production of indole-3-lactic acid (ILA), a breastmilk tryptophan metabolite, which acts through the aryl hydrocarbon receptor (AHR) mRNA expression and suppressed lipopolysaccharide (LPS)-induced IL-8 in premature intestinal enterocytes [98,99]. It also exerts its anti-inflammatory activity using a toll like receptor (TLR-4) as a mediator to inhibit IL-1β-induced IL-6 secretion and downregulates IL-1 receptor-associated kinase 2 (IRAK-2) mRNA expression in H4 cells [100], a common adapter protein associated with susceptibility to early-onset asthma [101]. Supplementation with B. subspecies infantis strain EVC001 is considered safe and tolerable by exclusively breastfed infants [102], and results in decreased production of IL-1β over the 60 days postnatal [103]. *B. infantis* strain R0033 supplementation exerts anti-inflammatory effects as indicated by an increase in the IL-10/IL-12 ratio, while the increase of the TNF-α/IL-10 ratio demonstrates a pro-inflammatory effect in infants following ingestion of *Lactobacillus helveticus* (*L. helveticus*) strain R0052 [104].

*B. longum* subsp. *infantis*, *B. bifidum*, *B. longum* subsp. *longum* and *B. breve* strains produce higher levels of ILA than adult-type and non-HRB strains [105], and an aromaticlactate dehydrogenase (LDH) gene from these strains is responsible for the production [106], suggesting ILA production by these strains may contribute to the inhibition of pro-inflammatory cytokines in asthma. The B. *bifidum* strain B536 isolated from infant feces has been found to display an inhibitory effect on LPS-induced IL-10 secretion [107]. The *B. bifidum* strains NCC189, S16, S17 [108] and Bif3 [109] exert inhibitory effects on LPS-stimulated NF-κB activation and mRNA/protein expression of IL-8 and TNF-α incolon adenocarcinoma cell line (HT-29) cells. The *B. longum* strain Lon4 inhibits LPS-TNF-α-dependent IL-8 mRNA/protein expression levels in HT-29 cells [109]. Probiotic supplementation with *B. longum* strain BB536 in healthy full-term infants enhances Th1/Th2 immune response through reducing the ratio of IFN-*γ*/IL-4 and the number of IFN-*γ* secretion cells [110]. Treatment of HT-29 cells with *B. breve* strain Bre10 suppresses LPS and TNF-α-induced IL-8 mRNA and protein expression levels [109]. The *B. breve* strain M-16V colonizing the breastfed infant gut is regarded as safety [111], and exerts anti-inflammatory effects on allergic diseases after a single supplementation [111,112], or in combination with *B. infantis* M-63 and *B longum* BB536 [113], including both prenatal and postnatal periods [114].The probiotic *B. breve* strain M-16V and galacto/fructo-oligosaccharide (GOS/FOS) prebiotic mixture supplemented with fortified milk increase the relative abundances of *Bifidobacterium* spp. [115]. Probiotic supplementation with *B. breve* in preterm infants increases the production of serum transforming growth factor (TGF-β) levels, and enhances the deca-pentaplegic homolog3 (Smad3) mRNA expression for TGF-A signaling molecules [116]. Treatment with microRNA (miR-744) mimic results in a significant decrease in the proliferation rate of bronchial epithelial cells in severely asthmatic children, and an increase of TGF-β1 mRNA abundance in these cells via regulating the Smad3 signaling pathway [117], suggesting that *B. breve* may enhance regulatory TGF-β1 and produce anti-inflammatory effects in asthma. Taken together, these findings suggest that various probiotic strains of the species Bifidobacterium *longum* subsp. *infantis*, *bifidum*, *longum* subsp. *longum* and *breve* have the potential to alleviate gut inflammatory responses in infants, which may reduce the risk of developing allergic asthma.

#### 5.2.2. *Lactobacillus* spp.

Lactobacillus are key members of the gram-positive facultative anaerobe rod-shaped lactic acid bacteria (LAB) with low GC content [94,118], which belong to class Bacilli within phylum Firmicutes [94,119], and are considered to be D(−), L(+) and DL-Lactic acid producers [120]. The Mediterranean diet (MED), characterized by consumption of plant protein, unsaturated fat/low fat, fiber/resistant starch and polyphenols has been found to increase the gut abundance of lactobacillus [96]. Early infancy colonization with *Lactobacillus* spp. reduces the risk of allergic asthma later in life [121]. Reduced abundances of *Lactobacillus* spp. are found in infants born to pregnant women with asthma [122]. *Lactobacillus* spp. and strains may be considered as potential therapeutic strategies against asthma in early infancy. *Lactobacillus* spp. isolated from breast milk showed anti-adhesion activity against enteric pathogens on intestinal epithelial cells and immunomodulation properties by suppressing IL-8 expression [123]. Specific isolated *Lactobacillus* strains from the infant feces display potential anti-inflammatory properties *in vitro*. *L*. *fermentum* BioE LF11 and *L. plantarum* BioE LPL59 showed anti-inflammatory properties through inhibiting LPS-stimulated IL-6 secretion [124]. *L*. *gasseri* 4M13 and *L. rhamnosus* 4B15 exhibit the highest cholesterol-lowering and anti-inflammatory activities by reducing cholesterol uptake from the intestine, and suppressing IL-6, IL-1β, IL-10 and mRNA expression of cytokine genes in the LPS-stimulated HT-29 cells [125]. *L. rhamnosus* JL-1 exerts a strong anti-inflammatory ability to attenuate infant gut inflammation induced by LPS stimulation through downregulation of the mRNA and protein expression of inflammatory genes (IL-6, IL-1β and TNF-α) in Caco-2 cells [126]. *L. paracasei* CNCM I-4034 demonstrates high anti-inflammatory activity as indicated by reduced pro-inflammatory cytokines (IL-6, chemokines and TNF-α) secretion in DCs [127]. One prospective study showed that early gut colonization with *Staphylococcus aureus* (*S. aureus*) in the presence of *L. rhamnosus GG* is associated with decreased production of IL-4 and IL-10 at two years of age compared to *S. aureus* alone, suggesting that *L. rhamnosus GG* exerts anti-inflammatory effects by suppressing *S. aureus*-induced pro-inflammatory cytokine production, and may modulate the immune responses and the risk of asthma [128]. These findings suggest that *Lactobacillus* strains as potential probiotics may contribute to reducing asthma by their ability to exert its immunomodulatory effect in the infant gut against LPS-induced inflammation.

#### 5.2.3. *Bacteroides* spp.

*Bacteroides* are Gram-negative anaerobic rod-shaped motile bacteria [94,96,129], which belong to the phylum *Bacteroidetes* [119]. The relative abundance of *Bacteroides* has been noted to increase with the intake of MED and animal fat/protein [96]. *Bacteroides* strains belonging to the species *B. fragilis*, *B. thetaiotaomicron* and *B. vulgatus* exert anti-inflammatory effects and immune regulation, which may contribute to their beneficial action in reducing asthma. *B. fragilis* exerts its immunomodulatory effects on cytokine production [130], and modulates the immunogenicity of LPS, which triggers pro-inflammatory cytokine production involved in gut inflammation [131]. *B. fragilis* has the ability to produce surface capsular polysaccharide A (PSA) by host DCs, which activates the TLR2 signaling pathway required to induce CD4^+^ Foxp3^+^ T_regs_ in vivo [131,132], and CD39^+^ Foxp3^+^ T_regs_ in vitro with marked suppression of LPS-induced monocyte TNFα [133]. Studies specifically investigating the effect of *B. fragilis* on immune development and asthma are limited. In a prospective study, colonization with *B. fragilis* at age 3 weeks is found to be associated with asthma at the age of 3 years. However, the study has the limitation that no tested fecal samples are obtained from mothers, so a complete recovery of *B. fragilis* strains in infants is unknown [134]. Another prospective study showed that early gut colonization with *B. fragilis* in high amounts at age 1 week and 1 month after birth is associated with suppressing TLR4 mRNA expression and LPS-induced IL-6 andC-C motif chemokine ligand (CCL4) at age 12 months [135], suggesting such bacterium may influence immune development and reduce the risk of asthma in later life.

*B. thetaiotaomicron* in vitro has the ability to produce regulatory IL-10 and protective IL-6 mediated by nanosized outer membrane vesicles (OMVs) [136]. *B. thetaiotaomicron* elicits anti-inflammatory activity in vitro through inducing nuclear export of the NF-κBrelaxedaspartate-auxotrophic (RelA)-PPARγ complex incolon carcinoma cell line (Caco-2) cells, causing downregulation of NF-κB-driven pro-inflammatory genes such as TNFα [137]. The NF-κB pathways regulate T-cell differentiation via regulation the expression of pro-inflammatory genes in asthma, particularly those encoding IL-6 and TNF-α [138], suggesting that *B. thetaiotaomicron* may have a potential role in this regulation. *B. vulgate* has been acknowledged for their anti-inflammatory effect in vitro. The isolated LPS*_Bv_* and Lipid A*_Bv_* from *B. vulgatus* have proven anti-inflammatory activity as evidenced by their stimulating low levels of TNF-α, IL-6, C-X-C motif chemokine ligand (CXCL-8) production and NF-κB activation compared to that of *Escherichia coli* (*E. coli*) LPS [139]. This suggests that *B. vulgatus* may have a potential immunemodulatory role in the context of asthma by reducing LPS-induced inflammation. Further RCTs are needed to highlight the anti-inflammatory and immune-modulatory effect of *B. thetaiotaomicron* and *B. vulgatus* in neonatal intestinal epithelial cells via regulation of asthmatic cytokine gene expression.

#### 5.2.4. *Enterococcus* spp.

*Enterococcus*, a gram positivefacultative anaerobe bacterium of the class Bacilli within the *Firmicutes* phylum [94,96,119], and with low GC content [94,140], is among the earliest LAB colonizers of the intestine in the first few days after birth [67,141]. Diets enriched in wheat bran and whole grains act as a prebiotic that may result in significantly increased fecal abundance of enterococci [142]. Delayed colonization with Enterococcus in early life is associated with subsequent development of allergy [143], indicating that early gut Enterococcus colonization could significantly influence immune development and reduce the risk of asthma. Specific *Enterococcus* strains may exhibit anti-inflammatory and regulatory effects in reducing asthma. A recent experimental study has found that the *E. faecalis* strain BioE EF71 isolated from infant feces resulted in suppressing LPS-induced IL-6 secretion [124]. The *E. faecalis* strains (EC1, EC3, EC15 and EC16), which are isolated from infant feces, have been found to suppress tumor receptor associated factor (TRAF6), TLR3, TLR4 and TLR9 in Caco-2 and HT-29 cells [144], through suppressing the activation of p38 mitogen-activated protein kinase (P38 MAPK) and the c-JUN NH2-terminal kinase (JNK) signaling pathways [145]. Inhibition of these signals would enhance TGF-β1 activation-induced connective tissue growth factor (CTGF) mRNA expression in asthmatic airway smooth muscle (ASM) cells and lung fibroblasts [146]. RCTs have shown evidence that p38 MAPK inhibition resulted in anti-inflammatory effects when used in combination with dexamethasone (synthetic glucocorticoid) on glucocorticoid receptor (GR) target gene expression through a significant reduction of LPS-induced IL-6 secretion associated with asthma in lung cells [147,148]. This suggests that *E. faecalis* may be involved in regulating inflammatory responses in infant lung cells and producing anti-inflammatory effects in asthma through immune-signaling pathways.

#### 5.2.5. *Streptococcus* spp.

*Streptococcus*, mainly represented by *S. salivarius* and *S. *thermophilus** GC-poor species [149], are the most predominant Gram-positive facultative anaerobe LAB of the class Bacilli within the phylum Firmicutes detected in both breast milk and the infant gut [150,151,152,153,154]. Consumption of high levels of unsaturated and low-fat diets appears to increase gut abundance of *Streptococcus* [96]. An imbalance in the gut microbiota between *Streptococcus* and other bacterial taxa appear to contribute to the development of allergic diseases in infants [155]. Specific probiotic *Streptococcus* strains have been shown to exert anti-inflammatory properties, which may help to reduce the risk of asthma in infants. A recent RCT has shown that infant formulas that included a combination of bioactive compounds (fermented formula) produced by *S. thermophilus* O65/*B. breve* C50 and prebiotic oligosaccharides increased fecal secretory immunoglobulin A (SIgA) levels in breastfed infants [156]. *S. *thermophilus** strain BioE ST107 isolated from infant feces exhibits immunomodulatory effects through an attenuation of the LPS-stimulated increase in IL-6 levels [124]. In an experiment study, the *S. thermophilus* strain St065 isolated from breast-fed infant feces produces active bacterial metabolites (mainly pepsin/trypsin), which inhibited LPS-induced NF-κB activation and IL-10 secretion [107]. This suggests that probiotic strains of *S. thermophilus* attenuate an LPS-induced pro-inflammatory response and may be an adjuvant treatment of asthma.

#### 5.2.6. *Blautia* and *Ruminococcus* spp.

*Blautia* spp., and in particular, *B. wexlerae, B. luti, B. producta, B. hansenii* and *B. faecis*, which belong to the family Lachnospiraceae within the phylum *Firmicutes* [94], constitute one of the most predominant *Clostridium coccoides* group (clade XIVa *Clostridium*) in infant feces [157,158,159]. Gut colonisation with *Blautia* spp. is influenced by early life dietary patterns. A recent prospective study found that unspecified *Blautia* spp. are positively associated with processed meat/savoury snacks/milk and inversely associated with meat/fish and eggs/beans over the first year of life [115], suggesting that *Blautia* may have the capacity to grow in foods rich in simple CHO and SAT.Most members of misclassified Ruminococci, including *R. obeum*, *R. luti*, *R. bromii, R. productus* and *R. gnavus*, which belong to the family Lachnospiraceae and clade IV *Clostridium*, are reclassified within the genus *Blautia* [160,161].

Studies on the gut colonization with *Blautia*/*Ruminococcus* and asthma risk in early infancy are limited. A recent prospective study revealed that individuals with a persistent gut colonization of *Blautia* and *Ruminococcus* are at high risk of asthma over the first year of life, though few infants display a significantly depleted in *Blautia*. It also shows that delayed gut microbial diversification observed in infants at high risk for asthma is remedied by daily supplementation with *L. rhamnosus* GG for 6 months [162]. An in vitro study revealed that the depletion of *B. wexlerae* and *B. luti* in obese children not only contributed to intestinal inflammation, but also to the development of insulin resistance and subsequent risk of diabetes. The study showed that gut colonization with these species is significantly less abundant in obese children (aged 5–14 years) than in lean counterparts. The study also showed that *B. luti* DSM 14534 and *B. wexlerae* F15 strains in lean children exert anti-inflammatory effects through reducing the ratio of IFN-γ/IL-4 and TNF-α/IL-4 in peripheral blood mononuclear cells compared to the effects of *B. vulgatus* strain BAC-CCC-2. This suggests that such species may help to reduce inflammation and improve glucose metabolism in lean children [163]. A prospective twin cohort study showed that high fecal abundance of *R. gnavus* drives Th2 allergic asthmatic responses through increased secretion of colonic IL-25 and IL-33. However, it is not clear whether *R. gnavus* induces Th2 allergic responses through mechanisms of allergy development or its genes. The study suggests that *R. gnavus* should be considered as a therapeutic target for the treatment of asthma [164]. Another prospective study revealed that a low relative abundance of *Ruminococcus* in infant feces at 1 month of age is associated with TLR2-induced IL-6/TNF-α and subsequent eczema at 6 months [165]. Given that *Blautia* and *Ruminococcus* spp. have been identified as butyrate-producing bacteria, further studies investigating its anti-inflammatory effects on immune-linked asthma in early infancy are needed.

#### 5.2.7. *Faecalibacterium* *Prausnitzii*

*F. prausnitzii* is a Gram positive, anaerobe, rod-shaped and non-motile bacterium [96], which belongs to the family *Ruminococcaceae* and clade IV *Clostridium* within the phylum *Firmicutes* [131,166]. Consumption of animal protein and SAT increase the relative gut abundance of *F. prausnitzii* [96]. *F. prausnitzii* exerts an anti-inflammatory effect through inhibiting NF-κB activation and IL-10 production in asthmatic children [167]. *F. prausnitzii* in vitro stimulates intestinal function, as evident by the attenuated expression of NF-κB, IL-1β and TNF-α in Caco-2 cells [168]. An experimental study showed that *F. prausnitzii* induces human colonic DCs to prime CD4^+^ and CD39^+^ expression secreting IL-10 and IL-27, which are known as molecules that play a key role in Foxp3^+^-T_reg_ generation and regulate asthma. *F. prausnitzii* in vitro suppresses LPS-induced expression of TNF-α/IL-12 and the JNK/MAPK signaling pathway activated by TLR2/6 ligands [169]. This suggests that *F. prausnitzii* inhibits LPS-mediated inflammation and induces colonic T_reg_ differentiation, which may help to reduce allergic asthma. Given the fact that children with increased risk of asthma had a low abundance of *F. prausnitzii* in early infancy [25,97,162], further studies are needed to investigate the role of gut *F. prausnitzii* in infancy, and elucidate the mechanisms underlying the *F. prausnitzii* asthma interaction, and understand the means by which such bacteria could reduce the risk of allergic asthma in early childhood.

Table 1 shows studies addressing the anti-inflammatory effect of SCFA-producing bacteria in reducing the production of inflammatory mediators involved in the pathogensis of asthma.

## 6. Concluding Remarks

The effects of VLCKD during pregnancy and lactation on the infant gut microbiota, and the mechanisms of its potential action in the treatment of asthma are still not fully understood. The VLCKD induces changes to the gut microbiota composition of pediatric patients, suggesting that the gut microbiota may hold a significant therapeutic potential to reduce asthma. The infant gut microbiota may be influenced by maternal VLCKD during pregnancy. The VLCKD during lactation may also directly influence the infant gut microbiota by influencing the breast milk microbiota. The VLCKD may lead to dramatic changes in epigenetic markers such as histone modification and DNA methylation. Epigenetic changes are also influenced by the KBs particularly βOHB, which exerts anti-inflammatory effects in vivo and in vitro. Adherence to VLCKD, a regimen low in CHO, high in fat and with moderate protein intake, leads to nutritional ketosis, which results in increased KBs production.

The SCFAs as epigenetic metabolites produced by gut microbiota belongining to the *Firmicutes* phylum may play a key stabilizing role for underpinning VLCKD-infant gut microbiota interactions, which may in turn reduce asthma risk. Butyrate can regulate host immune homeostasis to exert anti-inflammatory effects by inhibiting the production of asthma-related inflammatory cytokines. Other intermediate metabolites such as lactate produced by intestinal *Bifidobacterium* and LAB is crucial in VLCKD-infant gut microbiota interactions, where it acts with acetate as substrates for butyrate production.

SCFA-producing bacteria play an immune-modulating role in reducing asthma. Maternal bacterial species produce SCFAs as key metabolites exerting anti-inflammatory properties through passing the infant intestinal barrier. Several studies support the use of specific probiotic strains in preterm infants, which can influence SCFAs production and downregulate asthma-related inflammatory cytokines and chemokines.

In conclusion, SCFAs are key microbial metabolites that mediate the relationship between VLCKD during pregnancy and lactation, and the infant gut microbiota. The VLCKD regimen, including sources of dietary fiber, fats (high in PUFA, moderate in MUFA and low in SAT) and plant-based protein, may influence SCFA-producing bacteria in gut microbiota, and therefore, lead to an anti-inflammatory state and a decreased risk of asthma. High-quality clinical trials are needed before VLCKD can be recommended for pregnant and lactating women. Further large prospective cohort studies to monitor the changes in maternal gut microbiota composition during pregnancy and lactation following VLCKD are needed. This highlights the importance of monitoring the side effects and evaluating the effects of VLCKD on the infant gut microbiota composition or diversity with the aim of reducing asthma, which is associated with gut dysbiosis.

## Figures and Tables

**Table 1:** Summary of In Vitro Studies Demonstrating the Therapeutic Implications of SCFA-Producing Bacteria for Reducing Asthma in Infants.

Microorganism/s	Type	Anti-Inflammatory Effects in Asthma	References
*B. infantis*	Probiotic	↓ LPS-induced IL-8, ↓ IL-1β-induced IL-6	[98,99,100]
*B. infantis* EVC001, R0033	Probiotic	↓ IL-1β, ↑ anti-inflammatory ratio (IL-10/IL-12)	[103,104]
*B. bifidum* NCC189, S16, S17, Bif3, B536, *S. thermophilus* St065	Probiotic	↓ LPS-induced IL-10 &NF-κB, ↓ TNF-α	[107,108,109]
*B. longum* Lon4, BB536	Probiotic	↓ LPS-induced TNF-α, ↓ IFN-*γ*/IL-4	[109,110]
*B. breve* Bre10	Probiotic	↓ LPS &TNF-α-induced IL-8	[109]
*L*. *fermentum* BioE LF11, *L. plantarum* BioE LPL59, *E. faecalis* BioE EF71, *S. thermophilus* BioE ST107	Probiotic	↓ LPS-induced IL-6	[124]
*L*. *gasseri* 4M13, *L. rhamnosus* 4B15, *L. rhamnosus* JL-1	Probiotic	↓ LPS-induced IL-6, IL-1β, IL-10, TNF-α	[125,126]
*L. paracasei* CNCM I-4034	Probiotic	↓ IL-6, TNF-α	[127]
*L. rhamnosus GG*	Probiotic	↓ IL-4, ↓ IL-10	[128]
*B. fragilis*	Commensal	↓ IL-6, CCL4	[135]
*E. faecalis* EC1, EC3, EC15, EC16	Probiotic	↓ TNF receptor-associated factor 6 (TRAF6), TLR3, TLR4, TLR9	[144]
*S. thermophilus* O65/*B. breve* C50	Probiotic	↑ SIgA	[156]
*B. luti* DSM 14534, *B. wexlerae* F15	Probiotic	↓ IFN-*γ*/IL-4, ↓ TNF-α/IL-4	[163]
*F. prausnitzii*	Commensal	↓ IL-10, NF-κB	[167]

(↓) decrease, (↑) increase; table derived from reference [170].

## Abbreviations

ACA	Acetoacetate
Acetyl-CoA	Acetyl-coenzyme A
AHR	Aryl hydrocarbon receptor
AMPK	AMP-activated protein kinase
ANP	Atrial natriuretic peptide
βCT	β-ketoacyl-CoA transferase
βDH	βOHB dehydrogenase
BMI	Body mass index
βOHB	β-hydroxybutyrate
Caco-2	Colon carcinoma cell line
CCL	C-C motif chemokine ligand
CHO	Carbohydrate
CTGF	Connective tissue growth factor
CXCL	C-X-C motif chemokine ligand
DCs	Dendritic cells
DKA	Diabeticketoacidosis
ER	Endoplasmic reticulum
FADH2	Flavin adenine dinucleotide
FFAs	Free fatty acids
FOS	Fructo-oligosaccharide
Fox	Forkhead box
GATA3	GATA binding protein *3*
GC	Guanine-plus-cytosine
GOS	Galacto-oligosaccharide
GPCRs	G-protein coupled receptors
GR	Glucocorticoid receptor
HDACs	Histone deacetylases
HEK293	Human embryonic kidney 293
HRB	Human-Residential Bifidobacteria
HT-29	Colon adenocarcinoma cell line
IL	Interleukin
IFN	Interferon
IkB	Inhibitor of kappa B
IKK	Kappa B kinase
ILA	Indole-3-lactic acid
ILC2s	Group 2 Innate lymphoid cells
IRAK-2	IL-1 receptor-associated kinase 2
JNK	c-JUN NH2-terminal kinase
Kac	Histone/lysineacetylation
Kbhb	β-hydroxybutyrylation
KBs	Ketone bodies
KD	Ketogenic diet
Kme	Histonemethylation
LAB	Lactic acid bacteria
LCDs	Low carbohydrate diets
LDH	Lactate dehydrogenase
LPS	Lipopolysaccharide
MCTI1	Monocarboxylate transporter 1
MED	Mediterranean diet
miR	MicroRNA
MnSOD	Manganese superoxide dismutase
Mt2	Metallothionein 2A
MUFA	Monounsaturated fatty acid
NADH	Nicotinamide adenine dinucleotide
NAPA	Nitrate reductase catalytic subunit
NF-κB	Nuclear factor-κB
NLRP3	Leucine-rich-containing family, pyrin domain-containing-3
OMVs	Nanosized outer membrane vesicles
ORMDL3	ORM (yeast)-Like protein isoform 3
PSA	Polysaccharide A
PPARγ	Peroxisome proliferator-activated receptor gamma
PUFA	Polyunsaturated fatty acid
P38 MAPK	p38 mitogen-activated protein kinase
RelA	Relaxedaspartate-auxotrophic
RCTs	Randomized controlled trials
ROS	Reactive oxygen species
SAT	Saturated fatty acid
SCFAs	Short-chain fatty acids
SERCA	Sarco-endoplasmic reticulum Ca^2+^ pump
SIgA	Secretory immunoglobulin A
Smad	Deca-pentaplegic homolog
ST2	Stimulation-expressed gene 2
TCA	Tricarboxylic acid
TGF	Transforming growth factor
TLR	Toll like receptor
TNF-α	Tumor necrosis factor
TRAF	Tumor receptor associated factor
T_regs_	Regulatory T cells
TSLP	Thymic stromal lymphopoietin
UPR	Unfolded-protein response
VLCD	Very low-calorie diet
VLCKD	Very low-calorie ketogenic diet
